# Monitoring Mortality in Forced Migrants—Can Bayesian Methods Help Us to Do Better with the (Little) Data We Have?

**DOI:** 10.1371/journal.pmed.1001887

**Published:** 2015-10-20

**Authors:** Peter Heudtlass, Niko Speybroeck, Debarati Guha-Sapir

**Affiliations:** Institut de Recherche Santé et Société (IRSS), Université catholique de Louvain, Brussels, Belgium

## Abstract

In crisis situations, data are scarce—Peter Heudtlass and colleagues explore ways of best understanding the extra risk of death borne by forced migrants.

**Summary Points**

- Forced migrants are an extremely vulnerable population, but reliable data on all-cause mortality are chronically scarce.
- Almost all available estimates are based on data that are collected by humanitarian organizations and often suffer a lack of precision and potential publication bias.
- With two examples from South Sudan and Iraq, we demonstrate how these shortcomings can make audiences jump to conclusions too quickly, based on too little data. We propose an approach borrowed from clinical research to mitigate this problem.
- There are ongoing efforts by humanitarian organizations to improve the quality of data. Methodological innovations like this could complement these by providing tools to better deal with small samples of data coming from multiple agencies.

## Introduction: Forced Migrants, All-Cause Mortality, and the Difficult Quest for Reliable Data

The global number of forced migrants is currently the highest since the Second World War [1]. This is a major concern to public health: lack of access to safe water, food, sanitation, and inadequate shelter causes substantial increases in all-cause morbidity and mortality in displaced populations [2]. The number of victims due to infectious diseases can be higher than the direct death toll due to violence, even in particularly brutal conflicts such as the 2003 genocide in Sudan’s Darfur region [3].

However, the true extent of the problem is poorly documented, as there is no systematic health data collection in forced migrants. Deaths often take place beyond the reach of standard health data sources and therefore slip through the net of vital registration systems or large nation-wide health surveys, a key public health resource in developing countries. Disturbingly, the evidence is weakest for the most vulnerable populations, such as internally displaced persons (IDPs), who are displaced within their home country but do not benefit from the same international protection rights and support as refugees do. For instance, researchers were unable to include IDPs in recent national surveys in Colombia and Ethiopia because of security concerns [4,5].

In such settings, humanitarian organizations are often the only source of data on all-cause mortality. They routinely conduct household surveys and use data on migration, births, and deaths in random samples of their beneficiaries to retrospectively estimate all-cause mortality [6]. These surveys are done at a subnational level and are typically only representative for a few tens of thousands of people: from populations in camps to groups of villages and districts. As part of a growing effort to make these data more comparable and reliable, sampling methods, survey tools, and outcome measures were standardized in recent years [7].

The aim of such ad hoc assessments is to determine the severity of a humanitarian crisis. A group of major humanitarian organizations (including the International Federation of Red Cross and Red Crescent Societies, the International Medical Corps, Save the Children, Oxfam, and World Vision) have come together in the Sphere project to agree on thresholds (Table 1) for crude death rates (CDRs, all ages) and an under-five death rate (U5DR, only children under five years of age) that indicate public health emergencies: generally, twice of what is considered to be a normal death rate in a population is defined as a situation that requires immediate international relief operations [8,9]. These thresholds are now widely used in humanitarian practice.

**Table 1:** Baseline mortality levels and emergency thresholds by region.

	CDR Baseline	CDR Emergency Threshold	U5DR Baseline	U5DR Emergency Threshold
	(all values in deaths/10,000/day)			
Sub-Saharan Africa	0.41	0.8	1.07	2.1
Middle East and North Africa	0.16	0.3	0.27	0.5
South Asia	0.22	0.4	0.46	0.9
East Asia and Pacific	0.19	0.4	0.15	0.3
Latin America and Caribbean	0.16	0.3	0.15	0.3
Central and Eastern European Region/Commonwealth of Independent States and Baltic States	0.33	0.7	0.14	0.3
Industrialized Countries	0.25	0.5	0.03	0.1
Developing countries	0.22	0.4	0.44	0.9
Least-Developed Countries	0.33	0.7	0.82	1.7
World	0.25	0.5	0.4	0.8

Source: Sphere Handbook: Humanitarian Charter and Minimum Standards in Disaster Response, 2011 [9]

Small-scale mortality assessments are often part of routine surveys on a range of health outcomes and process variables and are intended mainly for internal use, in activities such as planning, monitoring, and evaluation of relief efforts. Even though these are usually shared with stakeholders and the local humanitarian community, they only occasionally find their way to the wider public. Crucially, whether or not the results of such assessments are disseminated is essentially in the hands of the humanitarian organizations themselves. Being not only service providers but also advocates for their beneficiaries and, more importantly, being largely dependent on private and institutional donations, humanitarian organizations have a potential conflict of interest. As a result, evidence of high mortality might be more likely to be disseminated than evidence of low mortality.

This problem is often aggravated by a lack of statistical power [10]. Mortality is not necessarily the main outcome of the study, which might have had an impact on the sample size chosen—often sufficient for a different outcome but insufficient to assess mortality with an acceptable level of precision. Sometimes, the sample size targets are missed because data collectors cannot access all sampled households. Furthermore, sample sizes are often chosen to give precise estimates of CDRs at the total population (all ages) level, but death rates are still also reported for subgroups (particularly children under five). In consequence, many death rate estimates lack precision, making it difficult to tell whether exceptionally high (or even low) observed death rates are not just the result of chance.

## Mortality Estimates Based on Too Little Data: Two Examples from South Sudan and Iraq

In July 2012, the nongovernmental organization (NGO) Médecins Sans Frontières (MSF) conducted a survey in a population of mostly refugees originating from the Sudanese Kordofan province and settled in Yida, South Sudan [11]. MSF claimed it observed a death rate of 3.98 deaths per 10,000 children under five years of age per day, meaning that children were dying at a rate almost four times higher than in “normal” sub-Saharan settings. The study results were reported by international newspapers, including *The Guardian* [12]. However, the uncertainty around this estimate was extraordinary large, as reflected by the wide 95% confidence interval (CI) of 0.55 to 7.39 deaths/10,000 children/day. Is this conclusive evidence of an acute humanitarian emergency or a statistical outlier?

In clinical research, a similar situation can arise when looking at interim results of clinical trials: early estimates, based on a relatively small number of observations, might suggest that a new treatment works. But do researchers see a true treatment effect or just a statistical anomaly? In 2004, Spiegelhalter proposed a pragmatic approach, based on Bayesian statistics, to deal with these situations [13]. Essentially, Bayesian statistics allow researchers to express a prior belief and use Bayes’ theorem to evaluate new data and update this belief. Many argue this is a more adequate way of learning from small samples of data than using the so-called frequentist statistical methods. Elements of Bayesian methods are increasingly used in health research and also in emergency needs assessments [14]. Spiegelhalter’s idea concerns in particular the formulation of the prior belief and is summarized briefly as follows: whenever you see a high (treatment) effect, put yourself in the shoes of a skeptical audience and evaluate the data based on the prior belief that there is actually no effect. If the Bayesian estimate (a synthesis of the prior belief and the new data) still shows a strong effect, enough evidence has been accumulated to convince even a skeptical audience, and the trial could (and possibly even should) be stopped early.

Since the publication of Spiegelhalter’s piece, the uptake of Bayesian methods in clinical research has continued to increase [15]. Admittedly, early stopping of clinical trials for effectiveness/futility based on pessimistic/enthusiastic priors is still rare (as is early stopping of clinical trials in general), although not unheard of [16]. The key contribution of Spiegelhalter’s paper is to use a set of priors that represents the whole spectrum of prior beliefs. Compared to an approach in which the researcher formulates a single prior distribution that reflects his or her own best knowledge or belief, this is arguably more objective and actually rather conservative. It has the potential to make Bayesian methods more acceptable to people whose concern is that such methods introduce too much subjectivity into the analysis, and therefore we believe it can be useful in humanitarian mortality assessments too. The concept is also under consideration in other innovative fields of medical research such as living systematic reviews [17].

We argue that this reasoning can also be applied to the example of the extraordinary high death rate reported by MSF: a skeptical audience could arguably assume that the death rate in Yida is similar to the average death rate in sub-Saharan countries, allowing for some degree of uncertainty. A Bayesian analysis of the death rate in the Yida camp then yields an estimate of 1.85 deaths/10,000 children/day (95% CI 0.75–3.08; see Table 2). This death rate is still elevated and of concern but far below MSF’s estimate of 3.98. It also signifies that a skeptical audience, based on their own expectations and the data that MSF has provided, would not be convinced that the internationally agreed emergency threshold of 2.1 deaths/10,000 children/day has been crossed.

**Table 2:** Under-five death rate in Yida refugee camp, June/July 2012.

	Mean	95% Confidence Interval**
Classical estimate (as reported by MSF)	3.98 deaths/10,000 children/day	0.55–7.39
Bayesian estimate* (using a “skeptical” prior)	1.85 deaths/10,000 children/day	0.75–3.08

* The Bayesian estimate is based on a conjugate beta-binomial model. The “skeptical” prior has a mean of 1.07 (sub-Saharan mortality baseline reference), and the 0.95 percentile is 2.1 (this means we assume the skeptical audience believes there is a 5% chance that mortality levels exceed the emergency threshold).

** The Bayesian “confidence interval” is the highest density interval (HDI) of the posterior distribution.

There is another common and related kind of error when making an inference from small samples: concluding from a small sample that mortality levels are not of concern when in reality they are. Therefore, according to Spiegelhalter, the researcher should, when facing a particularly low estimate based on small samples, take the point of view of an audience that does expect to see a high result.

In October 2014, the United Nations Children's Fund (UNICEF) and the Kurdistan Ministry of Health (MoH) assessed all-cause mortality in IDPs in the Duhok governorate in Iraq [18]. They estimated the CDR in the total population was 0.64 deaths/10,000 persons/day, which is more than twice the emergency threshold for Middle Eastern countries (0.3). However, the death rate in children under five years appeared to be comparably low, with 0.46 deaths/10,000 children/day (95% CI 0.18–1.15). This is somehow surprising, as U5DRs are typically higher than CDRs. Even though this could partly be explained by conflict-related violence, which causes deaths mostly among adults, a worried humanitarian audience could think this is instead due to the small sample, a result of chance. If we assume this audience believed the U5DR is, say, at least as high as the CDR, this leads to a Bayesian estimate of 0.55 deaths/10,000/day (Table 3). Thus, the available evidence is too weak to convince an audience who was expecting fairly high mortality levels that mortality among children in Duhok is below the emergency cut-off point of 0.5 deaths.

**Table 3:** Under-five death rate among internally displaced persons in Duhok governorate, Iraq, September/October 2014.

	Mean	95% Confidence Interval**
Classical estimate (as reported by UNICEF/MoH Iraq)	0.46 deaths/10,000 children/day	0.18–1.15
Bayesian estimate* (using a “high mortality” prior)	0.55 deaths/10,000 children/day	0.21–0.88

*The Bayesian estimate is based on a conjugate beta-binomial model. The “high mortality” prior has a mean of 0.64, and the 0.05 percentile is 0.27 (this means we assume there is still a 5% chance that mortality levels are at or even below baseline levels).

**The Bayesian “confidence interval” is the HDI of the posterior distribution.

The Bayesian CIs in both examples still include the relevant thresholds, and further data collection would be advisable. However, the intervals are narrower, and the difference between the Bayesian point estimate (the posterior mean) and the frequentist point estimate is substantial: 1.85 instead of 3.98 deaths/10,000 children/day in the South Sudan example and 0.55 instead of 0.46 in the case of Iraq. In addition, Bayesian inference also allows us to directly compute quantities of interest such as the probability of death rates exceeding twice the baseline, taking respective priors and the observed data into account: in the South Sudan example, this probability is 30.9%, and in the Iraq example 56.9%. These probabilities could be compared against a predefined threshold (e.g., 95%) that defines what amount of evidence is sufficient to trigger an international response.

Details on how we derived the above estimates can be found in the Supporting Information (S1 Text and S2 Text).

## Conclusion and Discussion

Forced migrants are a blind spot in global health datasets. Even though there are strong reasons to believe that they carry a considerable burden of preventable mortality, available evidence is patchy and scarce and does not come from independent sources. Death rate estimates are based on small samples of households and are prone to be influenced by chance. Because data collectors may have a conflict of interest, high death rates in particular are more likely to reach the media. This risks distorting the allocation of already limited funds in a humanitarian system that seeks to ground decision-making on hard evidence. The approach presented here can be seen as a tool to put data from underpowered mortality assessments into perspective, “adding to, rather than replacing, standard statistical techniques,” as suggested by Spiegelhalter in his original paper [13]. It can help prevent jumping to conclusions too quickly, based on too little data: if the evidence from observed data is very weak because the sample size is low, as in the two examples above, the Bayesian estimate will be clearly dominated by the prior beliefs, and the data have little influence on it. On the other hand, the more available observations are, the less influential the prior beliefs will be. It can be seen as a formal way of assessing how much (and sometimes how little) power the data at hand have to change the beliefs or knowledge of different audiences. Bayesian inference has the advantage that it uses more available information and allows this information to be combined with the observed data to estimate mortality rates. By using prior distributions, more information is used, and 95% credible intervals of posterior distributions are narrower than 95% CIs of frequentist point estimates. Bayesian credible intervals should always be reported alongside the priors used, as well as frequentist CIs. Also, in order to maximize the acceptability of this approach, a good strategy would be to advocate that researchers should spell out their priors before data collection.

Moreover, mortality assessments should be designed in a way that the estimates are precise enough to convince all relevant audiences, irrespective of any prior beliefs. The two examples above were underpowered studies, and it is important to bear in mind that in sufficiently large samples, any prior belief will be dominated by the data and Bayesian estimates are equivalent to the classical frequentist estimate.

But the reality is that conducting health research in humanitarian emergencies is extremely challenging: because of issues with security and access and extreme scarcity of resources such as staff, transportation, and time, it is much more difficult to recruit the targeted sample size than in other fields of health research. Because of these challenges, inadvertently underpowered studies such as the two above are the norm rather than the exception: 95% of the mortality estimates in the Complex Emergency Database (holding more than 3,000 surveys and one of the largest publicly accessible repositories) have an inadequate precision according to the definition of adequate precision by Prudhon and Spiegel [9] (the width of the 95% CI should be within 50% of the point estimate). Simply discarding the results of these studies would be an immense waste of data and resources in situations in which both are particularly scarce. Even though the Bayesian approach described here cannot replace careful research design and conduct, it is a pragmatic and affordable tool for decision makers to make better use of the great number of underpowered studies.

A Bayesian approach with a set of priors allows best- and worst-case scenarios to be explored but at the same time prevents overly alarmist estimates that are based on too little data, such as in the South Sudan example above. Overstatement and alarmism can have a role in advocacy but are rather counterproductive in scientific research, as in the long run, they undermine its credibility. For instance, an extreme mortality estimate based on a large-scale cluster survey in Iraq [19] has prompted a heated debate and has meant all mortality studies are now under suspicion.

From a scientific point of view, we would welcome more systematic and independent data collection or larger surveys to monitor mortality in forced migrants, but we are skeptical as to whether this is feasible in practice. Health data collection in emergencies is highly decentralized, which reflects the geographic spread of affected populations, the diversity of their needs, and the multitude of humanitarian agencies addressing these needs. In many crises, local staff of a few agencies are the last and only ones to have access to affected populations. There is probably no single institution that has sufficient financial resources, expertise, credibility, and access that could take on this task at a global scale. However, the humanitarian community is aware of the need to base their interventions on solid evidence and is working towards this is in their own interest. Indeed, there are successful interagency collaborations to standardize health indicators (Sphere project) and survey methodologies (Standardized Monitoring and Assessment of Relief and Transitions [SMART] methodology) and to share data with other organizations, researchers, and the public (Complex Emergency Database [CEDAT]) [7,9,20].

The cause of monitoring mortality and health in forced migrants might best be helped by gradually enhancing the capacities developed by these initiatives. Nevertheless, because of the very nature of humanitarian emergencies, decision makers will probably always have to deal with partial information and small samples of data. Methodological innovations such as the Bayesian approach proposed here can help better dealing with the specific challenges of data collection and decision-making in emergencies. From a more political perspective, the next step could be a commitment by humanitarian agencies to register all planned mortality and other health assessments in emergencies. This would, at a relatively low cost, improve the quality and usefulness of such studies by reducing publication bias.

## Supporting Information

S1 Text Technical notes. (PDF)

S2 Text R script to derive Bayesian estimates. (TXT)

S3 Text Summary Points in French. (PDF)

## Abbreviations

CDR: crude death rate
CEDAT: Complex Emergency Database
CI: confidence interval
HDI: highest density interval
IDP: internally displaced person
MoH: Ministry of Health
MSF: Médecins Sans Frontières
NGO: nongovernmental organization
SMART: Standardized Monitoring and Assessment of Relief and Transitions
U5DR: under-five death rate
UNICEF: United Nations Children's Fund
