# Supplementary material for: Monitoring Mortality in Forced Migrants—Can Bayesian Methods Help Us to Do Better with the (Little) Data We Have?
Source: PLoS Med. 2015 Oct 20;12(10):e1001887. doi: 10.1371/journal.pmed.1001887 (PMC4617888; doi:10.1371/journal.pmed.1001887)
Supplement: S1 Text — (PDF) [file pmed.1001887.s001.pdf]

S1 Text. Technical notes

1. South Sudan example
    a. Reported data and derivations

The following data was extracted from the survey report:[1]

- Under five death rate (U5DR, point estimate): 3.98
- 95% Confidence interval: 0.55, 7.39
- total population: 4685 people of which 22.2% are children under five years of age
- recall period: 35 days

Based on above figures and in absence of a reported number, we assume a mid-period population at risk of 1040 children under five years of age.

We use this data to derive the number of under five deaths:

$$U5\ Deaths\ = \frac{U5DR}{10000} * (recall\ period * mid - period\ U5\ population\ at\ risk)$$

Cluster sampling was used which inflates the estimates' variance by a certain factor, the design effect. We calculate the design effect as ratio of actual variance (derived from the reported confidence interval) over theoretical variance (assuming simple random sampling).

b. Formulation of prior

We numerically fit a beta distribution (two parameters: $alpha_{prior}$ and $beta_{prior}$) with a mean of 1.07/10000 and a 0.95 percentile of 2.1/10000 (see R code).

c. Derivation of posterior

The posterior in this conjugate beta-binomial model also has a beta distribution and its two parameters, $alpha_{posterior}$ and$beta_{posterior}$, can easily be derived analytically:

$$alpha_{posterior} = alpha_{prior} + U5\ deaths/design\ effect$$

$$beta_{posterior} = Person\ time\ spent\ at\ risk/design\ effect - U5\ deaths/design\ effect + beta_{prior}$$

The mean of this distribution is:

$$mean_{posterior} = alpha_{posterior}\ /(alpha_{posterior} + beta_{posterior})$$

The 95% confidence interval reported is a Bayesian credible interval. We calculated the Highest Density Interval (HDI, using the r package binom.confint) which has the desirable property that at any point in this

[1] http://reliefweb.int/sites/reliefweb.int/files/resources/Full%20Report_887.pdf

interval, the probability density is higher than at any point outside of the interval. Mortality is modelled in units of deaths per day and the estimates of mean and credible interval are multiplied by 10000 to be reported in units of per 10000 per day.

2. Iraq example
    a. Reported data and derivations

The following data was extracted from the survey report:[2]

- Under five death rate (U5DR, point estimate): 0.46
- 95% Confidence interval: 0.18, 1.15
- Under five (U5) deaths: 7

We can use this data to derive the person-time spent at risk:

$$Person\ time\ spent\ at\ risk = 1/(U5DR/10000/U5\ deaths)$$

Cluster sampling was used which inflates the estimates' variance by a certain factor, the design effect. The design effect is not reported, but using numerical optimisation methods (see R code), we can determine a design effect such that the Wilson score 95% confidence interval for the proportion $observed\ deaths/design\ effect$ over $(recall\ period * mid-period\ U5\ population\ at\ risk)/design\ effect$ is very close to the reported 95% confidence interval.

    b. Definition of prior

We numerically fit a beta distribution (two parameters: $alpha_{prior}$ and $beta_{prior}$) with a 0.05 percentile of 0.27/10000 and a mean of 0.64/10000 (see R code).

    c. Derivation of posterior

We used the same approach as outlined above for the South Sudan example.

[2] http://reliefweb.int/sites/reliefweb.int/files/resources/Duhok_NutSurveyOct23_Final.pdf
