# Supplementary material for: Monitoring Mortality in Forced Migrants—Can Bayesian Methods Help Us to Do Better with the (Little) Data We Have?
Source: PLoS Med. 2015 Oct 20;12(10):e1001887. doi: 10.1371/journal.pmed.1001887 (PMC4617888; doi:10.1371/journal.pmed.1001887)
Supplement: S3 Text — (PDF) [file pmed.1001887.s003.pdf]

S3 Text. Summary Points in French

- Les "migrants forcés" sont une population extrêmement vulnérable, mais des données fiables sur leur mortalité (toutes causes confondues) sont chroniquement rares.
- La majorité des estimations disponibles sont basées sur des données recueillies par des organisations humanitaires et souvent, elles souffrent d'un manque de précision et d’un biais de publication.
- A travers deux exemples au Soudan du Sud et en Irak, nous démontrons comment ces lacunes peuvent menés le grand public à des conclusions hâtives, basées sur trop peu de données. Nous proposons d'utiliser une approche de recherche clinique pour palier à ce problème.
- Les organisations humanitaires tentent améliorer la qualité de leurs données. Les innovations méthodologiques comme celle présentée ici pourraient compléter ces efforts en fournissant des outils pour mieux traiter de petits échantillons de données provenant de différentes organisations humanitaires.
